# Repeated Activation of a CS-US-Contingency Memory Results in Sustained Conditioned Responding

**DOI:** 10.3389/fpsyg.2013.00305

**Published:** 2013-05-30

**Authors:** Els Joos, Debora Vansteenwegen, Bram Vervliet, Dirk Hermans

**Affiliations:** ^1^Faculty of Psychology and Educational Sciences, University of Leuven, Leuven, Belgium

**Keywords:** conditioning, human learning, CS-US-contingency, rehearsal, post-acquisition processing, conditioned suppression

## Abstract

Individuals seem to differ in conditionability, i.e., the ease by which the contingent presentation of two stimuli will lead to a conditioned response. In contemporary learning theory, individual differences in the etiology and maintenance of anxiety disorders are, among others, explained by individual differences in temperamental variables (Mineka and Zinbarg, 2006). One such individual difference variable is how people process a learning experience when the conditioning stimuli are no longer present. Repeatedly thinking about the conditioning experience, as in worry or rumination, might prolong the initial (fear) reactions and as such, might leave certain individuals more vulnerable to developing an anxiety disorder. However, in human conditioning research, relatively little attention has been devoted to the processing of a memory trace after its initial acquisition, despite its potential influences on subsequent performance. Post-acquisition processing can be induced by mental reiteration of a conditioned stimulus-unconditioned stimulus (CS-US)-contingency. Using a human conditioned suppression paradigm, we investigated the effect of repeated activations of a CS-US-contingency memory on the level of conditioned responding at a later test. Results of three experiments showed more sustained responding to a “rehearsed” CS+ as compared to a “non-rehearsed” CS+. Moreover, the second experiment showed no effect of rehearsal when only the CS was rehearsed instead of the CS-US-contingency. The third experiment demonstrated that mental CS-US-rehearsal has the same effect regardless of whether it was cued by the CS and a verbal reference to the US or by a neutral signal, making the rehearsal “purely mental.” In sum, it was demonstrated that post-acquisition activation of a CS-US-contingency memory can impact conditioned responding, underlining the importance of post-acquisition processes in conditioning. This might indicate that individuals who are more prone to mentally rehearse information condition more easily.

## Introduction

In classical conditioning, a learning experience is often considered to end when the conditioning stimuli are no longer present. This is based on the fact that conditioning refers to the contingent presentation of an originally neutral stimulus (conditioned stimulus, CS) together with a biologically relevant unconditioned stimulus (US), resulting in the CS becoming a signal for US-onset and thus evoking a conditioned response (CR) during subsequent presentations (Bouton, 2007). This CR can be decreased or eliminated by non-reinforced presentations of the CS, a procedure called “extinction.” It is generally assumed that conditioning comprises both learning and memory: the learning of a CS-US-contingency builds up a memory (“encoding”), which is stored (“consolidation”) and can be reactivated upon future confrontations with that CS (“retrieval”). The strength of the CR is a function of these three processes.

Most conditioning research is focused on the encoding phase (which comprises the actual learning) and much less on consolidation and retrieval. However, the latter phases may have major effects on long-term conditioning. For instance, in the case of Pavlovian conditioning, human participants may mentally reflect upon the conditioning experience by repeatedly reactivating either the CS-representation, the US-representation, or the entire experience (CS-US-contingency memory). This repeated thinking about a negative experience might be akin to repetitive thought processes such as worry and rumination, as will be discussed in more detail shortly. The current research aimed to investigate the role of individual differences in such repetitive thought on the strength of conditioned responding.

Current evidence suggests that repeated reactivation of the conditioning memory results in higher CRs at a later test compared to conditions that do not include such active post-acquisition processing. First, the impact of US-rehearsal has been investigated by Davey and colleagues (Jones and Davey, 1990; Davey and Matchett, 1994). After conditioning, participants in the experimental group were asked to rehearse the US whenever the word “think” was presented on the screen, while control participants rehearsed either a non-aversive event or an unrelated aversive event. It was demonstrated that participants who rehearsed the US after acquisition retained a skin conductance response (SCR) during subsequent CS-presentations while this was not true for controls. Arntz et al. (1997) replicated this finding using SCR’s, but not when relying on anxiety ratings. Arguably, mental repetition of the US leads to stronger conditioned responding upon subsequent CS-presentations.

A second active post-acquisition procedure that could result in stronger conditioned responding is mentally reiterating the CS-US-*contingency*. As it is well-known that the contingency between the CS and the US is important in determining a CR, the procedure of repeated post-acquisition activation of the CS-US-contingency memory merits investigation as well. A preliminary indication of this effect can be found in a study by Yaremko and Werner (1974) who showed that repeatedly imagining a previously presented tone-shock-contingency elicited more pronounced electrodermal responses during subsequent extinction than imagining the same stimuli in an unpaired way. Imagining was cued by auditory presentations of the words “tone” and “shock.” Although not set up as a study about post-acquisition processing in conditioning (thus lacking appropriate control for acquisition strength), this study at least suggests that repeated mental activation of a CS-US-contingency impacts subsequent CRs. A second line of studies, performed in rabbits, provides only indirect support for a role of rehearsal in conditioning. Wagner and colleagues (e.g., Wagner et al., 1973; Terry and Wagner, 1975) investigated rehearsal as an explanatory mechanism for the fact that a US needs to be unexpected for CS-US-learning to occur. They suggested that rehearsal of conditioning events was crucial in conditioning. Furthermore, given that the rehearsal capacity of an organism is limited, they predicted (and showed) that a surprising event that would command rehearsal could interfere with the necessary rehearsal and thus with the learning of other CS-US-pairings. Based on these findings, Wagner (1981) developed the model of Standard Operating Procedures (SOP) which states that conditioned associations require the joint rehearsal of the representations of the CS and the US in memory.

It is surprising that these post-acquisition processes have received only little attention in human conditioning research, while they play a central role in the memory literature. For instance, rehearsal, a type of post-acquisition processing defined as the covert or overt repetition of information (Atkinson and Shiffrin, 1971) is studied extensively and is implicated as an important factor in most models of memory functioning (Anderson, 1999). It is well established that more rehearsal, both in frequency as in length of the rehearsal period, typically results in enhanced memory for the rehearsed information (Ebbinghaus, 1885/1913; Johnson, 1980). As conditioning relies on both learning and memory (Bouton and Moody, 2004), it seems obvious to study post-acquisition rehearsal processes in conditioning. In a first attempt to address this issue, we (Joos et al., 2012b) investigated the role of post-acquisition processing in fear learning, by examining the impact of rehearsing an aversive conditioned association (CS = picture; US = human scream) on subsequent fear responding. Fear responding to the picture-CS which was previously paired with the scream persisted in participants who rehearsed this contingency, but decreased in participants who had been asked to rehearse a different contingency. In the current manuscript, the role of post-acquisition processing is studied in a conditioned suppression paradigm that allows us to investigate whether the earlier findings in fear conditioning also apply in a more neutral contingency learning task. More importantly, giving it’s less time-consuming nature, this paradigm allows a more in depth analysis of the influence of rehearsal on conditioned responding using different rehearsal procedures (see below).

Besides the theoretical relevance of post-acquisition processes in conditioning, studying these processes is ecologically valid as well. In general, it is believed that some aspects of anxiety disorders are explained by conditioning processes (Rachman, 1991; Craske et al., 2006; Field, 2006), i.e., undergoing a conditioning experience (such as a car accident) can install subsequent fear reactions (such as driving phobia). However, large differences exist in whether or not an individual develops an anxiety disorder after such an (aversive) learning experience.

In contemporary learning theory, it is suggested that such individual differences in the etiology and maintenance of anxiety disorders could, among others, be explained by individual differences in temperamental variables, such as trait anxiety and behavioral inhibition (Levey and Martin, 1981; Mineka and Zinbarg, 2006; Mineka and Oehlberg, 2008; Joos et al., under review). However, we hypothesize that the differential tendency to engage in repetitive thought, such as worry or rumination, might also be an important factor in explaining differences in conditionability. Not only is trait anxiety highly associated with repetitive thought (e.g., Meyer et al., 1990), but it is also demonstrated that trait worry predicts the strength of fear acquisition (Otto et al., 2007; Joos et al., 2012c). Participants scoring higher on the Penn State Worry Questionnaire (Meyer et al., 1990) demonstrated enhanced fear learning.

As such, differences in the strength of a CR, could in part be explained by differences in how people process a learning experience when the conditioning stimuli are no longer present. Individuals might repeatedly reflect upon an aversive conditioning experience, which might be akin to repetitive thought processes such as worry and rumination (Watkins, 2008). The potential role of repetitive thought in anxious responding is supported by the fact that individual differences in worry and rumination correlate with anxious symptoms (e.g., Meyer et al., 1990; Segerstrom et al., 2000; Fresco et al., 2002; Muris et al., 2004; Ehring et al., 2011). Moreover, repetitive thought (worry and/or rumination) has even been shown to predict the level of anxiety or anxiety symptoms in prospective designs (Nolen-Hoeksema, 2000; Segerstrom et al., 2000; Calmes and Roberts, 2007; Hong, 2007; McLaughlin et al., 2007; Watkins, 2008). In sum, the more one engages in repetitive thought, the more anxiety symptoms are experienced. Hence, given the role of conditioning processes in anxiety, we believe that repeatedly thinking about a conditioning experience, as in worry or rumination, might prolong the initial (fear) reactions and as such, might leave certain individuals more vulnerable to developing an anxiety disorder.

Given these correlational findings, we wanted to investigate experimentally the influence of differential post-acquisition processing (i.e., mental rehearsal) on subsequent conditioned responding. To this aim, we modeled differences in repetitive thought by experimentally inducing repeated activation of a conditioning experience. In the present studies, we targeted post-acquisition processing of the CS-US-contingency, rather than activation of the CS- or the US-representation. In Experiments 1 and 2, participants were primed to rehearse the CS-US-contingency by presenting the CS and a verbal label referring to the US, in line with Yaremko and Werner (1974). This procedure allowed us to control the content of participant’s thoughts. Moreover, this cued rehearsal procedure might resemble repetitive thought in real-life. A cue activates both the mental representations of the CS and the US and the association between them, but the US is never directly experienced. This resembles cued recall of fear memory by real-life confrontations with the phobic stimulus (e.g., driving a car). Given that this rehearsal procedure might entail additional acquisition trials (due to visual presentation of the CS), the procedure is contrasted with a purely mental rehearsal procedure, as used by Davey and colleagues (Jones and Davey, 1990). As repetitive thought is often purely mental as well, this procedure might more closely resemble ruminative thinking, i.e., repetitive thought that is cued by intrusions or memories of the conditioning event. In general, we hypothesize that mental reiteration of a CS-US-contingency, in the absence of real US-presentations, results in more conditioned responding compared to when no repeated activation occurs. This repetitive mental evocation of the CS-US-contingency memory is further referred to as “rehearsal.”

We used a conditioned suppression paradigm, known as the Martians preparation, which has proven to be sensitive to a wide range of CS-US-contingency manipulations (for a review, see Franssen et al., 2010). This preparation was developed by Arcediano et al. (1996) to create a human analog for the conditioned suppression task used in animal conditioning. In such a task, the amount of suppression of an operant response serves as a behavioral measure of the strength of Pavlovian conditioning. As the Martians preparation is developed for use in humans, an instructed US is employed, rather than a biologically significant US. The task is set up as a computer game in which participants utilize their laser-gun (space bar) to shoot Martians. However, shooting during activation of the anti-laser shield (US) results in an inescapable invasion of Martians. Space scenes (CSs) predict the occurrence of the anti-laser shield. Learning is evident when participants refrain from bar pressing during a CS+. Using this preparation it was examined whether rehearsing a CS-US-contingency results in more conditioned suppression to a rehearsed CS+ than to a non-rehearsed CS+.

## Experiment 1

### Materials and methods

#### Participants

Participants were 42 volunteers aged between 18 and 53 years (*M* = 22.17, SD = 6.01). They participated in partial fulfillment of course requirements or were paid for their participation. The study was conducted in accordance with the requirements of the ethical committee of the faculty. All gave informed consent and were instructed that they could decline further participation at any time. They were uninformed about the purpose of the experiment and had no previous experience with the Martians preparation, apart from one participant who was excluded specifically for this reason. Three other participants were excluded due to problems during the procedure (talking and being distracted during bar pressing). The results for 38 participants (ages 18–27, *M* = 21.05, SD = 2.20; 31 women) were included in the data-analysis.

#### Stimuli and apparatus

All participants were tested individually. Participants responded using the space bar of the keyboard. The Martians preparation was implemented into a flexible Windows95 ™environment by Baeyens and Clarysse (1998), using Microsoft Visual C++ 5.0 and was recently adapted into MartiansV2 by Franssen et al. (2010).

**Figure 1:**
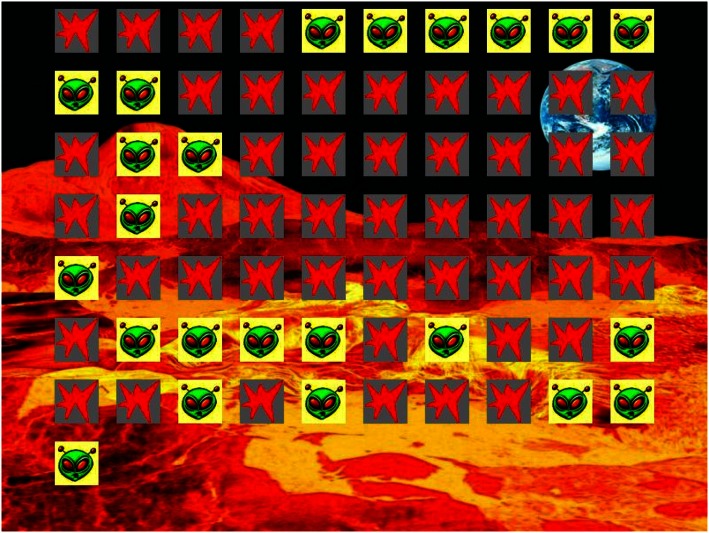
**Screenshot from the Martians preparation**. Depicted are Martians and explosions (when Martian is shot by pressing the space bar) against a CS-background picture of a space scene.

Background pictures of four multi-colored space scenes served as CSs and were counterbalanced across individuals. CS-duration was 1.5 s, but was extended to 3 s during crucial test trials. The US consisted of a 0.5 s white flashing screen (5 flashes at a rate of 10 flashes/s; interflash time = 50 ms) accompanied by a metallic sound played in continuous looping (73 dBa). All sounds were presented binaurally through headphones (Philips SHP 2000). The images of the Martians and the explosions that appeared after “shooting” a Martian were multi-colored stimuli measuring 50 × 50 pixels. A screenshot of the Martians preparation is presented in Figure 1.

#### Procedure

In the Martians computer game, participants have to shoot incoming Martians by pressing the space bar (operant behavior). A Pavlovian CS-US-contingency is superimposed on this operant task. The US is described as an anti-laser shield. Participants have to refrain from bar pressing during activation of this anti-laser shield because otherwise, an inescapable invasion of Martians follows. The Martians procedure typically consists of various phases. In the two experiments presented here, these were: pre-training phase, US-only phase, acquisition, and acquisition test phase, rehearsal phase, and rehearsal test phase.

During the *Pre-training phase*, participants learned to emit a regular pattern of operant responding (bar pressing). Martians landed on the screen in rows from left to right and from top to bottom at a rate of 4/s. A full screen consisted of 7 rows and 10 columns (inter-row distance = 20 pixels, inter-column distance = 20 pixels). If full, the screen rolled up in a continuous fashion to make room for new Martians. Participants learned to press the space bar at the same rate as the appearance of Martians (4/s). In that case, only explosions rather than Martians appeared. However, if participants barpressed at a higher rate, not all Martians were shot and explosions appeared occasionally. The task for the participant was to make as many explosions appear as possible. Neither CSs nor USs were presented during this phase.

During the entire experiment, instructions were given both orally and visually on the computer screen (for an overview of instructions, see Baeyens et al., 2001). Participants could practice the bar pressing behavior for 25 s (100 Martians) during which the experimenter gave oral guidance if needed. After this phase (and after the US-only phase, the acquisition/acquisition test phase and the post-rehearsal phase) visual feedback was provided in the form of hit percentage.

The purpose of the next phase, the *US-only phase* was to introduce the instructed US, represented by the so-called “anti-laser shield” (combination of a flashing screen and a metallic sound). During the US, Martians appeared at the same rate as before. In this phase no CSs were presented. Participants learned to refrain from bar pressing when the anti-laser shield was activated, since pressing the space bar during this period evoked an inescapable invasion of Martians. An invasion lasted for 5 s and consisted of the landing of “thousands” of Martians (at a rate of 20/s) accompanied by a new sound played in continuous looping (79 dBa). During this invasion, bar pressing was ineffective (no explosions appeared contingent upon bar pressing). The US-only phase entailed four trials. On average, the inter-trial interval lasted for 7.5 s (SD = 2.5 s). This was the case throughout the whole experiment. The first two trials were used by the experimenter to explain (a) what an anti-laser shield looks like and (b) what happens if one presses the space bar during the anti-laser shield. Throughout the following two trials, participants could practice avoiding bar presses during the US which was virtually impossible as the USs appeared unannounced.

The *Acquisition phase* entailed the introduction of Pavlovian CS-US-contingencies which were superimposed on the operant baseline task. Participants were instructed that indicators would appear (background pictures) that might predict the occurrence of the US. They had to learn to distinguish good (CS+) from bad (CS−) predictors. In case of a good predictor, participants had to refrain from bar pressing to avoid pressing during the anti-laser shield. In case of a bad predictor, this suppression behavior was undesirable as not pressing the space bar would result in the successful landing of numerous Martians.

The Acquisition phase included training with two different CS+s and two different CS−s. A CS+ was immediately followed by the US. A CS− was never followed by the US. Space scenes were counterbalanced between participants, serving either as the CS+ that would be rehearsed (CS+_R_) or that would not be rehearsed (CS+_NR_) or as one of both CS−‘s (CS−_A_ or CS−_B_). This resulted in 12 counterbalancing conditions. All CSs lasted for 1.5 s and were presented five times each, generating 20 (randomized) trials. For the CS+ trials, a 80% reinforcement schedule was used in order to obtain suboptimal conditioning.

The Acquisition phase was immediately followed by the *Acquisition Test phase*. This transition was not noticeable to participants. The Acquisition Test phase comprised one non-reinforced presentation of every CS. Trial order was again randomized. During this test phase, every CS was presented for 3 s instead of 1.5 s to allow a more accurate measurement of the suppression behavior. Throughout the Acquisition and the Acquisition Test phase, trials lasted for 7 s. During the ITI’s, the background screen was black. The light in the room was dimmed during these phases.

In the *Rehearsal phase*, the crucial manipulation was implemented. The goal was to prompt participants to mentally rehearse one of the CS-US-contingencies they had acquired in the previous phase. The CS+ that was part of the rehearsed CS-US-contingency is referred to as the rehearsed CS+. The other CS+ is called the non-rehearsed CS+. As a background for this differential mental rehearsal participants were asked to engage in a so-called attention training task that “could affect their future task performances.” More precisely, they were requested to focus their attention on one of the background pictures (CS+_R_) that was previously presented and to think about this background and how it co-occurred with the flashing anti-laser shield. When they noticed being distracted, participants had to gently refocus their attention on the background – anti-laser shield-compound. Participants were prompted to keep refocusing their attention whenever necessary. The training task was set up as a cover story to ensure rehearsal of both the CS and the US. The stimuli of the other CS-US-contingency were never presented during this phase. The background picture (CS+_R_) and the word “anti-laser shield” (in Dutch) were presented six times for 15 s, alternated with a 10-s black screen. This was done to repeatedly draw the attention of the participants to the screen. The attention training task lasted for 2 min 20 s and was conducted twice. After each training, participants rated how easy/difficult it was for them to focus (and keep focused) their attention on the background and the flashing anti-laser shield on a 21-point scale ranging from −100 (*very difficult*) to +100 (*very easy*) in steps of 10. In between both training tasks, a filler task consisting of two questionnaires was administered^1^.

After the Rehearsal phase the effects of mental rehearsal were monitored. Participants were redirected to the Martians computer task for the *Rehearsal Test phase*. Once more, the light was dimmed. Participants were instructed that the task was identical as before and that they were again expected to shoot Martians to stop them from invading Earth. They had to avoid bar pressing during the anti-laser shield and pay attention to the signals (CSs) to infer US-occurrence. No further instructions were given. However, when a participant asked whether the anti-laser shield would occur again, he/she was told that this possibility existed. The phase consisted of three blocks of one unreinforced presentation of the four CSs (CS+_R_, CS+_NR_, CS−_A_, and CS−_B_) to ensure a reliable assessment of conditioned responding after rehearsal. Within each block of four trials, trial order was randomized. Since testing occurred under extinction, the first test trial was the most crucial one as non-reinforced presentations might have reduced conditioned responding in the subsequent test trials. After the test phase, participants were thanked for their participation.

### Results

#### Manipulation check

After each training task, participants rated the difficulty of focusing their attention on a scale ranging from −100 (*very difficult*) to +100 (*very easy*). If participants reported being distracted during rehearsal, this might have influenced the quality of CS-US-rehearsal. The mean attention scores for the first and second attention training as well as the overall mean score for both tasks are presented in Table 1. As negative scores indicate difficulties to focus attention, we excluded for further analyses three participants who obtained a negative score averaged over both training tasks.

**Table 1:** **Mean attention score (and standard deviations) on the first and the second attention training task and average for both tasks, as a function of experimental group for Experiments 1, 2, and 3**.

		Attention training 1		Attention training 2		Average	
	
	
	
		*M*	SD	*M*	SD	*M*	SD
Experiment 1		58.05	28.77	40.34	37.72	49.20	30.47
Experiment 2	CS-US-rehearsal	56.25	33.73	32.50	39.92	44.38	31.84
	CS-rehearsal	66.25	18.61	29.58	40.59	47.92	24.54
	Visual rehearsal	46.30	40.11	34.44	35.99	40.37	36.90
Experiment 3	Mental rehearsal	23.08	50.89	21.53	45.67	22.31	45.59
	Control	49.60	37.47	27.60	49.69	38.60	41.72

#### Dependent variable

In conditioned suppression tasks like the Martians task, suppression of the operant response (bar pressing) serves as a measure of the strength of classical conditioning. Participants’ behavior is expressed in terms of suppression ratios (SRs) of the form *a*/(*a* + *b*), where *a* is the number of responses during the CS, and *b* the number of bar presses in an equal period of time immediately preceding CS-onset. This implies that a SR equaling 0.5 indicates no suppression at all, while a SR equaling 0 designates complete suppression of the operant response.

#### Acquisition test

**Figure 2:**
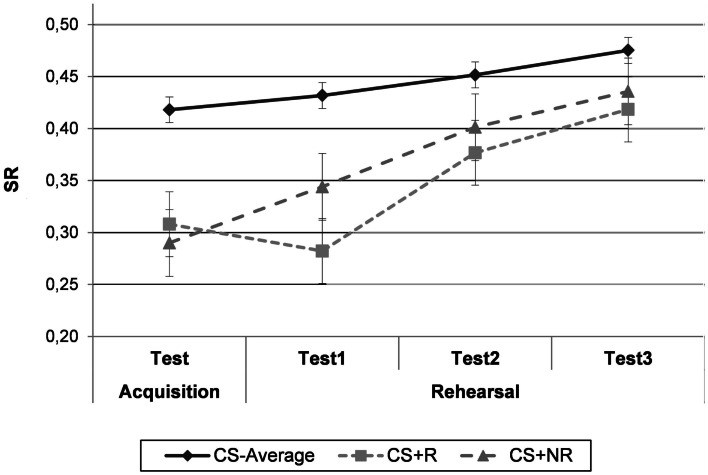
**Experiment 1**. Mean suppression ratio’s (SR) as a function of phase (acquisition test, rehearsal test 1, rehearsal test 2, and rehearsal test 3) and CS-type (CS−_Average_, CS+_R_, and CS+_NR_). CS−_Average_ = the average for both CS−‘s. CS+_R_ = rehearsed CS+, CS+_NR_ = non-rehearsed CS+. Error bars denote standard error.

Figure 2 displays SRs as a function of Phase and CS-type. Data from the Acquisition Test phase were analyzed using a repeated measures ANOVA with CS-type (CS−_Average_/CS+_R_/CS+_NR_) as within-subjects variable^2^. The main effect of CS-type was significant, *F*(2, 68) = 23.64, *p*  < 0.0001, MSE = 0.007, $\eta_{p}^{2}=0.41$, indicating successful differential acquisition. Planned comparisons indicated that the SRs for the CS+_R_ and the CS+_NR_ were significantly lower than the SR for CS−_Average_, *F*_CS+R_(1, 34) = 22.01, *p*  < 0.0001, MSE = 0.009; *F*_CS+NR_(1, 34) = 40.10, *p*  < 0.0001, MSE = 0.008. Given the interest in a differential effect between both CS+s after rehearsal, a non-differential level of conditioning for both CS+s is a prerequisite, which was fulfilled, *F*(1, 34) = 1.89, *p* = 0.18.

#### Rehearsal test

Figure 2 suggests that the rehearsal manipulation had an effect on the level of suppression to the CS+s at test. This was supported by a 2 (Phase: Acquisition test/Rehearsal test average) × 2 (CS-type: CS+_R_/CS+_NR_)-repeated measures ANOVA, including average SRs over three test trials (Rehearsal test average), which revealed a significant Phase × CS-type interaction, *F*(1, 34) = 5.53, *p*  < 0.05, MSE = 0.005, $\eta_{p}^{2}=0.14$. This indicates a different course of suppression over time to the rehearsed than to the non-rehearsed CS+. Planned comparisons at test demonstrated that the CS+_R_ evoked a stronger CR than the CS+_NR_, *F*(1, 34) = 4.26, *p*  < 0.05, MSE = 0.005. A decrease in conditioned suppression is present for both CS+s, but is stronger for the non-rehearsed CS+, *F*(1, 34) = 32.67, *p*  < 0.0001, MSE = 0.006, than for the rehearsed CS+, *F*(1, 34) = 5.63, *p*  < 0.05, MSE = 0.008.

Thus, the hypothesis about the effect of rehearsal on conditioned responding is supported. As the test phase comprised three trials which were conducted under extinction, the effect of rehearsal was also investigated for responding on the first test trial only. Using a 2 (Phase: Acquisition test/Rehearsal test 1) × 2 (CS-type: CS+_R_/CS+_NR_)–repeated measures ANOVA, it was shown that the crucial Phase × CS-type interaction was significant, *F*(1, 34) = 6.77, *p*  < 0.05, MSE = 0.009, $\eta_{p}^{2}=0.17$. Again, planned comparisons demonstrated that after rehearsal, the CS+_R_ evoked a stronger CR than the CS+_NR_, *F*(1, 34) = 5.69, *p*  < 0.05, MSE = 0.01. Moreover, conditioned responding to the non-rehearsed CS+ attenuated from acquisition test to rehearsal test 1, *F*(1, 34) = 6.03, *p*  < 0.05, MSE = 0.009, while this was not the case for the rehearsed CS+, *F*(1, 34) = 1.48, *p* = 0.23.

### Discussion

Experiment 1 was set up to test whether repeated activation of a previously acquired CS-US-contingency memory impacts conditioning effects to that CS in the long-term. The results clearly show stronger conditioned responding to the rehearsed CS+ as compared to the non-rehearsed CS+, indicating that mental reiteration of a CS-US-experience strengthens subsequent conditioned responding. An important observation is however that, rather than causing an increment in responding, rehearsal seems to sustain responding, while the absence of rehearsal results in decreased CRs. This pattern is further investigated in Experiment 3.

After demonstrating an effect of rehearsal, we wanted to explore the boundary conditions of this effect. As both CS+s were paired with the same US, the observed rehearsal effect cannot be attributed to rehearsal of the US alone. Indeed, US-rehearsal would elicit the same level of responding to both CS+s at test. However, at this point it is unclear whether repeated activation of the CS alone would result in increased conditioned responding as well. Indeed, it might be the case that the observed effect should be attributed to CS-rehearsal rather than to rehearsal of the CS-US-contingency. Experiment 2 was set up to investigate this possibility.

## Experiment 2

In addition to investigating whether the findings of Experiment 1 could be replicated, this experiment was conducted with the aim to extend these promising findings. More precisely, it was investigated whether mental reiteration of a CS alone results in increased or sustained responding as well, in which case the observed rehearsal effect in Experiment 1 could be attributed to CS-rehearsal.

That CS-rehearsal could increment responding has been shown in studies regarding sensitization or incubation. First, rehearsal of the CS might result in a sensitization of conditioned responding, i.e., an increase in responsiveness caused by the (covert) repetition of a stimulus (Groves and Thompson, 1970). As such, this might underlie a strengthening of the CR after CS-US-rehearsal. Second, in fear conditioning, it is proposed that short unreinforced presentations of the CS might result in an *increment*, rather than a decrement, in responding. Since Eysenck (1968) termed this phenomenon “incubation,” some studies have provided tentative support for this hypothesis (e.g., Rohrbaugh and Riccio, 1970; Rohrbaugh et al., 1972). Until now, little evidence exists that repeated CS-only presentations promote a *progressive increase* in CR strength (Nicholaichuk et al., 1982; Kaloupek, 1983). Most studies demonstrate that short duration CS-presentations evoke resistance to extinction, rather than incubation (Stone and Borkovec, 1975; Sandin and Chorot, 1989). A process of incubation, either defined as an increment in conditioned responding or a resistance to extinction, would result in more conditioned suppression to the rehearsed CS+ (as compared to the non-rehearsed CS+) after the rehearsal phase as well.

To test the impact of rehearsing the CS without reference to the US, two conditions were included. Besides the “CS-US-Rehearsal”-group, a replication of Experiment 1, this study comprised a control condition, “CS-Rehearsal.” Participants in this condition were requested to rehearse the CS, instead of the CS-US-contingency. Conversely, it is important to note that most learning theories would predict extinction, characterized by a *decrement* rather than an increment in CR, after unreinforced CS-presentations (Hermans et al., 2006). Given these conflicting predictions, it is important to include this control group. If conditioned responding is only sustained after CS-US-rehearsal and not after CS-rehearsal, the data from Experiment 1 cannot be ascribed to mental reactivations of the memory of the CS alone.

### Materials and methods

#### Participants

Fifty psychology students participated in return for course credits. All participants provided informed consent and were instructed that they could decline further participation at any time during the experiment. They were all uninformed about the purpose of the experiment. Two participants were excluded due to technical problems. The remaining 40 females and 8 males, aged 17–21 years (*M* = 18.13, SD = 0.74), were randomly assigned either to the condition “CS-US-Rehearsal” or the condition “CS-Rehearsal,” resulting in 24 participants in each condition.

#### Procedure

The same apparatus, software, and stimuli were used as in Experiment 1. For both conditions, the Pre-training phase, the US-only phase and the Acquisition and Acquisition Test phase were identical to those in Experiment 1. Only the Rehearsal phase differed between both experiments. Similarly, the aim of this phase was to evoke mental rehearsal of previously presented stimuli. However, while participants in one condition rehearsed a CS-US-contingency as in Experiment 1, participants in the other condition had to mentally rehearse a CS+, without reference to the US. Hence, participants in this condition were merely asked to focus their attention on one of the background pictures that was previously presented. As always, they were requested to gently refocus their attention when they noticed being distracted. As in Experiment 1, the attention training cover story was applied to obtain rehearsal. During the Rehearsal phase, the same parameters were used. More precisely, in the “CS-US-Rehearsal”-group, the background picture, and the word “anti-laser shield” (in Dutch) appeared on the screen six times. In the “CS-Rehearsal”-condition, only the background picture (CS+_R_) was presented, again for six times alternated with black screens.

The attention training was again executed twice and each training phase was followed by a short rating of the difficulty to focus their attention. The training phases were separated by the administration of three filler questionnaires. Upon completion of the Rehearsal phase, an unrelated computer task (causal learning task) was administered. Subsequently, participants were redirected to the first computer for the Rehearsal Test phase, which consisted of three blocks, each containing one unreinforced presentation of every CS.

### Results

#### Manipulation check

The mean attention scores for the first and second training task (see Table 1) did not differ between both conditions, *t*_1_(46) = 1.27, *p* = 0.21; *t*_2_(46) = 0.25, *p* = 0.80, nor did the attention score when averaged over both tasks, *t*(46) = 0.43, *p* = 0.67. As in Experiment 1, we excluded for further analyses the (two) participants who obtained a negative score when averaged over both training phases.

#### Acquisition test

**Figure 3:**
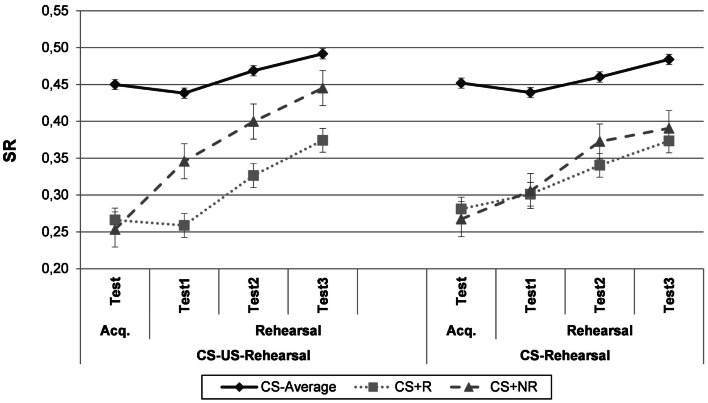
**Experiment 2**. Mean suppression ratio’s for the “CS-US-Rehearsal”-group, who rehearsed the CS-US-contingency, and the “CS-Rehearsal”-group, who rehearsed only the CS, as a function of phase (acquisition test, rehearsal test 1, rehearsal test 2, and rehearsal test 3) and CS-type (CS−_Average_, CS+_R_, and CS+_NR_). CS−_Average_ = the average for both CS−‘s. CS+_R_ = rehearsed CS+, CS+_NR_ = non-rehearsed CS+, Acq. Test = acquisition test. Error bars denote standard error.

Suppression ratio’s for each condition are presented in Figure 3, as a function of Phase and CS-type. Acquisition data were analyzed using a 2 × 3-repeated measures ANOVA with Group (“CS-US-Rehearsal”/“CS-Rehearsal”) as a between-subjects variable and CS-type (CS−_Average_/CS+_R_/CS+_NR_) as a within-subjects variable. Participants displayed differential conditioned responding to the CS+s as compared to the CSs. Overall, there was a main effect of CS-type, *F*(2, 88) = 73.83, *p*  < 0.0001, MSE = 0.007, $\eta_{p}^{2}=0.63$, but no significant Group × CS-type interaction, *F*(2, 88) = 0.04, *p* = 0.96, indicating no group differences in differential responding.

Planned comparisons confirmed that in the “CS-US-Rehearsal”-condition, the CS+_R_ and the CS+_NR_ generated significantly more conditioned responding than the CS−_Average_, *F*_CS+R_(1, 44) = 44.53, *p*  < 0.0001, MSE = 0.008; *F*_CS+NR_(1, 44) = 53.21, *p*  < 0.0001, MSE = 0.008. Participants demonstrated an equal amount of suppression for both CS+s, *F*(1, 44) = 0.55, *p* = 0.46. This indicates that no differences in responding existed between both CS+s before the onset of the rehearsal phase. Similarly, participants in the “CS-Rehearsal”-condition demonstrated more conditioned responding to the CS+_R_, *F*(1, 44) = 44.70, *p*  < 0.001, MSE = 0.008, and the CS+_NR_, *F*(1, 44) = 52.57, *p*  < 0.001, MSE = 0.008, than to the CS−_Average_. Again, no significant differences emerged between both CS+s, *F*(1, 44) = 0.45, *p* = 0.51.

#### Rehearsal test

The left panel of Figure 3 (“CS-US-Rehearsal”-condition) shows a data pattern that generally replicates Experiment 1. After rehearsal, the CS+_R_ evokes more conditioned responding than the CS+_NR_. In line with predictions, this data pattern is absent for the “CS-Rehearsal”-group. To investigate whether mentally rehearsing the CS-US-contingency or the CS alone differentially impacts subsequent CRs, a 2 (Group: “CS-US-Rehearsal”/“CS-Rehearsal”) × 2 (Phase: Acquisition test/Rehearsal test average) × 2 (CS-type: CS+_R_/CS+_NR_)-repeated measures ANOVA was conducted. As in the previous experiment, data of the three test trials were averaged to obtain a more reliable assessment. The Group × Phase × CS-type interaction failed to reach significance, *F*(1, 44) = 2.44, *p* = 0.13. However, after exclusion of the two participants who experienced difficulties focusing their attention during the *first* attention task rather than exclusion of those participants with a negative score when *averaged over both* tasks, the three-way interaction was marginally significant, *F*(1, 44) = 3.82, *p* = 0.057, MSE = 0.004. Although only marginally significant, the partial eta squared $\left(\eta_{p}^{2}\right)$ of.08 suggests that this interaction can be interpreted as a medium to large effect (Stevens, 2002).

Follow-up analyses using simple interactions showed that for the “CS-US-Rehearsal”-condition, the Phase (Acquisition test/Rehearsal test average) × CS-type (CS+_R_/ CS+_NR_) interaction was significant, *F*(1, 44) = 10.62, *p*  < 0.005, MSE = 0.005, $\eta_{p}^{2}=0.19$. Planned comparisons confirmed that the CS+_R_ produced significantly more conditioned suppression than the CS+_NR,_ *F*(1, 44) = 15.22, *p*  < 0.001, MSE = 0.004, at rehearsal test. Moreover, the decrease in suppression was significant for the CS+_NR,_ *F*(1, 44) = 45.74, *p*  < 0.0001, MSE = 0.005, but non-significant for the CS+_R_, *F*(1, 44) = 3.95, *p* = 0.05. For the “CS-Rehearsal”-condition, the overall Phase (Acquisition test/Rehearsal test average) × CS-type (CS+_R_/ CS+_NR_) interaction failed to reach significance, *F*(1, 44) = 1.32, *p* = 0.26. Rehearsing a CS+ alone does not seem to impact subsequent CRs to this CS. This conclusion is corroborated by planned comparisons showing no difference in SR between both CS+s at test, *F*(1, 44) = 0.87, *p* = 0.36. Responding to both CS+s decreased significantly from acquisition to rehearsal test, *F*_CS+R_(1, 44) = 5.84, *p*  < 0.05, MSE = 0.007; *F*_CS+NR_(1, 44) = 19.23, *p*  < 0.0001, MSE = 0.005.

Given that the Group × Phase × CS-type interaction failed to reach significance for the average SR over three test trials and given that rehearsal test 1 is probably the most valid test trial, the impact of rehearsal was also assessed for the first test trial only. A 2 (Group) × 2 (Phase: Acquisition test/Rehearsal test 1) × 2 (CS-type) ANOVA was conducted, which revealed a marginally significant three-way interaction, *F*(1, 44) = 3.98, *p* = 0.052, MSE = 0.007, $\eta_{p}^{2}=0.08$ (medium to large effect). Moreover, after exclusion of the two participants with difficulties to focus during the *first* attention task (instead of averaged over both tasks), this interaction was significant, *F*(1, 44) = 6.07, *p*  < 0.05, MSE = 0.006, $\eta_{p}^{2}=0.12$ (medium to large effect), providing support for the differential impact on conditioned responding of rehearsing a CS-US-contingency versus a CS alone.

Follow-up analyses targeting the data for the “CS-US-Rehearsal”-condition, yielded a significant Phase × CS-type interaction, *F*(1, 44) = 10.75, *p*  < 0.005, MSE = 0.007, $\eta_{p}^{2}=0.20$. Planned comparisons showed that the CS+_R_ produced significantly more suppression than the CS+_NR_ at rehearsal test 1, *F*(1, 44) = 13.48, *p*  < 0.001, MSE = 0.008. Comparable to Experiment 1, the SR for the CS+_R_ remained intact after rehearsal, *F*(1, 44) = 0.33, *p* = 0.57, while responding to the CS+_NR_ decreased from acquisition to rehearsal test 1, *F*(1, 44) = 13.24, *p*  < 0.001, MSE = 0.008. Taken together, these data replicate the finding of a strengthened CR to a CS after mental CS-US-rehearsal. Data for the “CS-Rehearsal”-condition showed that the Phase (Acquisition test/Rehearsal test) × CS-type interaction was non-significant, *F*(1, 44) = 0.29, *p* = 0.59, indicating that CS-rehearsal did not impact responding. Planned comparisons provided additional support for this conclusion by showing no difference in SR between both CS+s at rehearsal test 1, *F*(1, 44) = 0.03, *p* = 0.87. Unexpectedly, responding did not show a significant decrement between acquisition test and rehearsal test 1 for both CS+s, *F*_CS+R_(1, 44) = 0.53, *p* = 0.47; *F*_CS+NR_(1, 44) = 2.15, *p* = 0.15, as was the case for rehearsal test average.

In sum, it seems that while rehearsal of the CS-US-compound sustains conditioned responding to the rehearsed CS+ as compared to the non-rehearsed CS+, this is not the case when only the CS+ is rehearsed.

### Discussion

Experiment 2 was set up to test whether the effects of Experiment 1 were due to reactivations of the CS-memory or of the CS-US-memory. First, the results of the “CS-US-Rehearsal”-group in Experiment 2 replicated the findings of Experiment 1. As such, this study provides additional evidence that mental reiteration of a CS-US-contingency strengthens conditioned responding to that CS+ relative to responding to a non-rehearsed CS+. In addition, the results from the “CS-Rehearsal”-group showed no difference between the rehearsed and the non-rehearsed CS+ and as such, no evidence for sensitization or incubation of responding after CS-rehearsal was provided. This points to the conclusion that mental repetition of only the CS is not sufficient to produce the effect of Experiment 1 (sustained CRs at test). Because Experiments 1 and 2 also showed no effect on the CS+ that was conditioned to the same US but not rehearsed (CS+_NR_) in the CS-US-rehearsal groups, the observed effect should probably not be attributed to rehearsal of the US either. The main conclusion is that mental reiteration of a CS-US-contingency causes sustained conditioned responding, while reactivation of the CS or the US alone does not have this effect.

It is important to note that we cannot rule out the possibility that participants in the “CS-Rehearsal”-group might have also thought about the US during instructed CS-rehearsal. However, given the clear difference in instructions (and cueing on the screen) between both experimental groups, we believe that participants in the “CS-US-rehearsal”-condition at least thought more about the CS-US-compound than participants in the “CS-rehearsal”-condition.

An important question is to what extent our rehearsal manipulation might simply constitute additional acquisition trials. During the rehearsal phase, we presented the CS-picture and a verbal reference to the US. Although this procedure does not comprise experience with the actual US (sensory characteristics), it may produce additional learning of the mere CS-US-*contingency*. During “pure” rehearsal, these additional contingency experiences would be internally generated (thinking back of the co-occurrences of the CS and the US), whereas they were externally generated in Experiments 1 and 2. Moreover, repetitive thought does not only occur during presentations of the phobic stimuli (CS). Indeed, individuals often think about past conditioning experiences *in the absence* of the CS or US, so a purely mental rehearsal procedure would seem more akin to repetitive thought. Therefore, we conducted a third experiment, which included a new condition in which participants were prompted by a neutral signal to rehearse the CS-US-contingency in a purely mental way, i.e., without being primed by the visual presence of the CS and a verbal reference to the US. This procedure was in line with the paradigm used by Davey and colleagues (Jones and Davey, 1990; Davey and Matchett, 1994). A non-differential rehearsal effect in the visually aided rehearsal condition as in the purely mental rehearsal condition would indicate that the observed rehearsal effect should not be attributed to additional training during rehearsal.

A second important note is that both in Experiments 1 and 2 the rehearsal effect seems partly driven by a *decrease* in CR to the non-rehearsed CS+, rather than by an *increase* in CR to the rehearsed CS+. This decrement may reflect the natural course of conditioned responding over time; rehearsal would then prevent this spontaneous decrease in responding (see General Discussion). However, it is also possible that the CS-US-rehearsal trials primarily produce their effect by reducing responding to the non-rehearsed CS+, such that rehearsal of one CS-US-contingency interferes with responding to the other CS+, which was presented during the same learning phase. Such interference has been shown before, for instance in studies by Pineno and colleagues (Matute and Pineno, 1998; Pineño et al., 2000) demonstrating impaired responding to X when X+ training was followed by A+ training. Similarly, in our studies the decrease in CR to the non-rehearsed CS+ can be considered as the result of stimulus competition between elementally trained CSs evoked by mental rehearsal trials pairing the rehearsed CS+ and the US. A related memory phenomenon is *retrieval-induced forgetting*, which refers to the situation where retrieval of a subset of formerly studied material (e.g., CS+_R_ – US) causes subsequent forgetting of the non-retrieved material (e.g., CS+_NR_ – US; Bäuml et al., 2010, p. 1048). In a recent study by Ortega-Castro and Vadillo (2013), retrieval-induced forgetting was demonstrated using word pairs, where several cues predicted a common outcome. As such, rehearsal/retrieval of one CS-US-contingency might induce forgetting of the non-rehearsed contingency.

In order to evaluate this possibility, we included an extra control group in Experiment 3 who “rehearsed” an irrelevant picture-word pair after acquisition. This group will show the natural course of responding to a CS+ from acquisition to test. A significant decline in CRs for both CS+s in this group would indicate that the observed decrease in CRs to the non-rehearsed CS+ should not be attributed to interference.

## Experiment 3

Experiment 3 comprised three experimental groups. The first group, “Visual Rehearsal,” was largely a replication of the CS-US-rehearsal groups in the previous experiments, including visually aided CS-US-rehearsal. The second group, “Mental Rehearsal,” entailed a purely mental rehearsal procedure, without any visual guidance (except during instructions). Finally, participants in the “Control”-condition rehearsed an unrelated picture-word pair. We expected sustained suppression to the rehearsed CS+, but a decrease in responding to the non-rehearsed CS+ in both rehearsal groups. Moreover, a decline in CRs to both CS+s was expected in the “Control”-condition.

### Materials and methods

#### Participants

Eighty students participated either in return for course credits or as paid volunteer. They provided informed consent and were uninformed about the purpose of the study. Participants were randomly allocated to either the “Visual Rehearsal”-condition (*n* = 27), the “Mental Rehearsal”-condition (*n* = 27) or the “Control”-condition (*n* = 26). The data of two participants (from “Mental Rehearsal”-group and “Control”-condition) had to be excluded due to apparatus failure. The remaining 78 participants (63 women) had a mean age of 19.63 (SD = 2.46; range 17–34).

#### Procedure

The apparatus, software, and stimuli were again identical as in Experiment 1. Moreover, the same procedure was used, with only the Rehearsal phase differing from the previous studies. During this phase, all participants received instructions to rehearse the co-occurrence of two related stimuli, using the attention training cover story (with same parameters). In both rehearsal conditions (Visual/Mental Rehearsal), participants received the same instructions as in the CS-US-rehearsal conditions from the previous experiments asking them to focus their attention on one of the background pictures (CS+_R_) and how it co-occurred with the anti-laser shield. A slide with the CS and a verbal reference to the US was additionally presented on the computer screen to ensure that all participants were aware of the stimuli on which to “focus their attention.” While participants in the “Visual Rehearsal”-condition were presented with this CS-picture and a verbal reference to the anti-laser shield during the rehearsal phase, participants in the “Mental Rehearsal”-group saw only an exclamation mark, prompting them to a purely mental repetition of the conditioning stimuli. Participants in the “Control”-condition were also requested to focus their attention on a picture, a word and how they co-occurred. To ensure that they had equal visual experience with the CS-picture and the anti-laser shield as participants in the “Mental Rehearsal”-group, control participants were presented with a CS+ -picture and a verbal reference to the anti-laser shield as an example of a possible picture-word pair they could encounter in the following phase. Subsequently, it was further clarified that they had to focus on clouds and how these co-occurred with rain. A visual display of a picture of clouds and the word “rain” was presented during these instructions and during the following Rehearsal phase.

After each training phase, participants rated how difficult it was to focus their attention. In between both training phases, three questionnaires were administered. The Rehearsal phase and the Rehearsal Test phase were separated by an unrelated computer task (causal learning task).

### Results

#### Manipulation check

The attention score during the first and second attention training task and the score when averaged over both tasks (see Table 1) did not significantly differ between groups, as evidenced by the absence of an effect of group in several one-way ANOVAs with Group as between-subjects variable, *F*_training 1_(2, 75) = 2.89, *p* = 0.06, *F*_training 2_  < 1, *p* = 0.57, *F*_training 1+2_(2, 75) = 1.50, *p* = 0.23. As in Experiments 1 and 2, participants who obtained a negative attention score averaged over both training phases were excluded. This was the case for five participants (18.52%) in the “Visual Rehearsal”-condition, seven participants (26.92%) in the “Mental Rehearsal”-condition and four participants (16.00%) in the “Control”-condition.

#### Acquisition test

**Figure 4:**
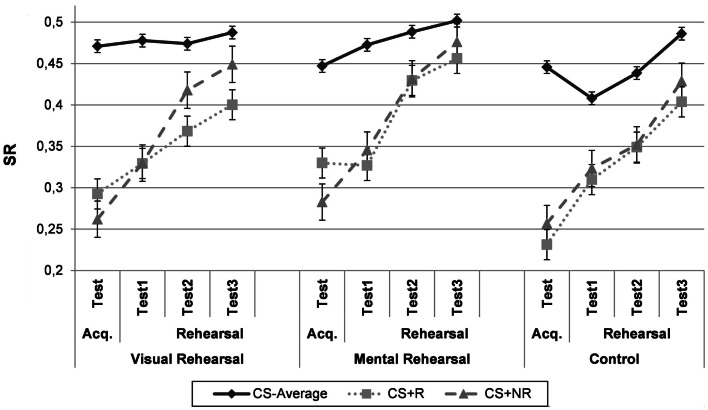
**Experiment 3**. Mean suppression ratio’s for the “Visual Rehearsal”-group, who rehearsed the CS-US-association with visual guidance, the “Mental Rehearsal”-group, who rehearsed the CS-US-contingency without visual guidance, and the “Control”-condition, who rehearsed an irrelevant picture-word pair, as a function of phase (acquisition test, rehearsal test 1, rehearsal test 2, and rehearsal test 3) and CS-type (CS−_Average_, CS+_R_, and CS+_NR_). CS−_Average_ = the average for both CS−‘s. CS+_R_ = rehearsed CS+,CS+_NR_ = non-rehearsed CS+, Acq. Test = acquisition test. Error bars denote standard error.

Figure 4 depicts SRs for each condition as a function of Phase and CS-type. As can be seen in the graph, participants show successful differential acquisition with higher SRs to the CS−_Average_ than to the CS+s. However, the level of responding to the CS+s after acquisition seems to differ according to the experimental group. This is corroborated using a 3 × 3-repeated measures ANOVA with Group (“Visual Rehearsal”/“Mental Rehearsal”/“Control”) as between-subjects variable and CS-type (CS−_Average_/ CS+_R_/ CS+_NR_) as within-subjects variable. This ANOVA yielded a main effect of CS-type, *F*(2, 118) = 125.25, *p* < 0.0001, MSE = 0.005, $\eta_{p}^{2}=0.68$, which was qualified by a significant Group × CS-type interaction, *F*(4, 118) = 2.76, p < 0.05, MSE = 0.005, $\eta_{p}^{2}=0.09$.

Further analyses showed that participants in the “Visual Rehearsal”-condition demonstrated less suppression to CS−_Average_ than to CS+_R,_ *F*(1, 59) = 58.40, *p* < 0.0001, MSE = 0.006, and CS+_NR_, *F*(1, 59) = 84.89, *p* < 0.0001, MSE = 0.006. Both CS+s evoked a non-differential amount of suppression, *F*(1, 59) = 2.44, *p* = 0.12. The same pattern was evident for participants in the “Control”-condition, with higher SRs to both CS+s than to CS−_Average,_ *F*_CS+R_(1, 59) = 80.53, *p* < 0.0001, MSE = 0.006; *F*_CS+NR_(1, 59) = 66.25, *p* < 0.0001, MSE = 0.006, and a non-differential level of responding to both CS+s, *F*(1, 59) = 1.61, *p* = 0.21. However, while participants in the “Mental Rehearsal”-condition again demonstrated more suppression to the CS+s than to the CS−_Average_, *F*_CS+R_(1, 59) = 21.80, *p* < 0.0001, MSE = 0.006; *F*_CS+NR_(1, 59) = 45.40, *p* < 0.0001, MSE = 0.006, they showed significantly more suppression to the CS+_NR_ than to the CS+_R,_ *F*(1, 59) = 5.01, *p* < 0.05, MSE = 0.004, which is unexpected given that the procedure was identical for both CS+s until then.

#### Rehearsal test

Figure 4 suggests a general decrease in CRs to both CS+s in the “Control”-condition and a smaller decrease in conditioned responding to the CS+_R_ than to the CS+_NR_ after CS-US-rehearsal in both rehearsal groups, which is in line with our hypotheses. This was supported by a 3 (Group: “Visual Rehearsal”/“Mental Rehearsal”/“Control”) × 2 (Phase: Acquisition test/Rehearsal test average) × 2 (CS-type: CS+_R_/ CS+_NR_)-repeated measures ANOVA. As before, data of the three test trials were combined. This analysis revealed a significant Phase × CS-type interaction, *F*(1, 59) = 7.46, *p* < 0.01, MSE = 0.003, $\eta_{p}^{2}=0.11$, that subsumed under a significant Group × Phase × CS-type interaction, *F*(1, 59) = 3.27, *p* < 0.05, MSE = 0.003, $\eta_{p}^{2}=0.10$, indicating that the course of responding to both CS+s was differentially influenced by the rehearsal manipulation depending on the experimental group.

The three-way interaction was further explored using simple interactions and planned comparisons. In the “Control”-condition, conditioned suppression significantly decreased at the same rate for both CS+s during rehearsal of an unrelated picture-word pair, *F*_CS+R_(1, 59) = 36.74, *p* < 0.0001, MSE = 0.004; *F*_CS+R_(1, 59) = 24.72, *p* < 0.0001, MSE = 0.005, as evidenced by a non-significant Phase × CS-type interaction, *F*(1, 59) = 0.25, *p* = 0.62. In contrast, in the “Visual Rehearsal”-condition, the Phase × CS-type interaction was significant, *F*(1, 59) = 7.62, *p* < 0.01, MSE = 0.003, indicating a stronger decrease in suppression for the CS+_NR_ than for the CS+_R_. Responding to both CS+s decreased from acquisition to test, but this decrease was larger for the CS+_NR,_ *F*(1, 59) = 39.28, *p* < 0.0001, MSE = 0.005, than for the CS+_R_, *F*(1, 59) = 13.73, *p* < 0.001, MSE = 0.004. These results point toward an effect of visually aided CS-US-rehearsal on subsequent CRs. Likewise, the Phase × CS-type interaction in the “Mental Rehearsal”-condition also reached significance, *F*(1, 59) = 6.03, *p* < 0.05, MSE = 0.003, with a smaller decrease in CRs for the CS+_R,_ *F*(1, 59) = 12.17, *p* < 0.001, MSE = 0.004, than for the CS+_NR_, *F*(1, 59) = 33.04, *p* < 0.0001, MSE = 0.005.

To investigate whether visually aided CS-US-rehearsal had a different impact on responding than purely mental CS-US-rehearsal, the Group (“Visual Rehearsal”/“Mental Rehearsal”) × Phase × CS-type interaction was assessed. This interaction failed to reach significance, *F*(1, 59) = 0.007, *p* = 0.94, suggesting that both forms of rehearsal influenced responding in the same way. Furthermore, given our hypothesis that the decline in responding to the CS+_NR_ after CS-US-rehearsal reflects a natural course of responding, the 3 (Group) × 2 (Phase) interaction was assessed for the CS+_NR_. This interaction was non-significant, *F*(2, 59 = 0.41, *p* = 0.66, indicating that responding to the CS+_NR_ decreases to the same extent after CS-US-rehearsal as after rehearsal of an irrelevant picture-word pair.

As the first test trial is considered the most valid one, the data were also analyzed when taking into account only the change in responding from post-acquisition to the first rehearsal test trial. A 3 (Group: “Visual Rehearsal”/“Mental Rehearsal”/“Control”) × 2 (Phase: Acquisition test/Rehearsal test 1) × 2 (CS-type: CS+_R_/ CS+_NR_)-repeated measures ANOVA revealed a non-significant Group × Phase × CS-type interaction, *F*(1, 59) = 1.25, *p* = 0.29. Further analyses for the three conditions separately revealed that for participants in the “Control”-condition, the Phase × CS-type interaction was not significant, *F*(1, 59) = 0.13, *p* = 0.72. Both CS+s evoked significantly less suppression at rehearsal test 1 than at acquisition test, *F*_CS+R_(1, 59) = 12.07, *p* < 0.001, MSE = 0.005, *F*_CS+NR_(1, 59) = 7.57, *p* < 0.01, MSE = 0.006, indicating that rehearsal did not impact responding. In the “Mental Rehearsal”-group, responding to the CS+_NR_ significantly decreased between acquisition and rehearsal test 1, *F*(1, 59) = 6.13, *p* < 0.05, MSE = 0.006, while no such decrease was present for the CS+_R_, *F*(1, 59) = 0.02, *p* = 0.90, suggesting sustained responding to the CS+_R_. The Phase × CS-type interaction was only marginally significant, *F*(1, 59) = 3.37, *p* = 0.07, MSE = 0.006. The same pattern emerged for participants in the “Visual Rehearsal”-group. Again, CRs significantly decreased for the CS+_NR_ during the rehearsal phase, *F*(1, 59) = 8.25, *p* < 0.01, MSE = 0.006, but not for the CS+_R,_ *F*(1, 59) = 2.78, *p* = 0.10. The Phase × CS-type interaction was however not significant, *F*(1, 59) = 0.86, *p* = 0.36.

Based on visual inspection of Figure 4, we tested whether the Phase × CS-type interaction differed between both rehearsal groups, given that the rehearsal effect on the first test trial seems more pronounced in the “Mental Rehearsal”-condition. The Group (“Visual/Mental Rehearsal”) × Phase × CS-type interaction failed to reach significance, *F*(1, 59) = 0.51, *p* = 0.48, indicating a non-differential effect of visually aided and purely mental rehearsal.

Given that the pattern of results in Figure 4 suggests a delayed effect of rehearsal in the “Visual Rehearsal”-group, we examined the change in responding between acquisition test and the final test trial for both CS+s using a 3 (Group) × 2 (Phase: Acquisition test/Rehearsal test 3) × 2 (CS-type) ANOVA. In line with the previous findings, the same rate of CR decrease was observed for both CS+s in the “Control”-condition, *F*_CS+R_(1, 59) = 47.89, *p* < 0.0001, MSE = 0.007, *F*_CS+NR_(1, 59) = 41.75, *p* < 0.0001, MSE = 0.007, with a non-significant Phase × CS-type interaction, *F*(1, 59) = 0.0003, *p* = 0.99. In contrast, in both rehearsal groups, suppression declined significantly stronger for the CS+_NR_, *F*_Mental Rehearsal_(1, 59) = 47.84, *p* < 0.0001, MSE = 0.007; *F*_Visual Rehearsal_(1, 59) = 51.78, *p* < 0.0001, MSE = 0.007, than for the CS+_R_, *F*_Mental Rehearsal_(1, 59) = 23.25, *p* < 0.0001, MSE = 0.007; *F*_Visual Rehearsal_(1, 59) = 19.57, *p* < 0.0001, MSE = 0.007, as evidenced by a significant Phase × CS-type interaction in both the “Mental Rehearsal,” *F*(1, 59) = 5.73, *p* < 0.05, MSE = 0.004, and the “Visual Rehearsal”-condition, *F*(1, 59) = 9.25, *p* < 0.005, MSE = 0.004. The overall Group × Phase × CS-type interaction was however only marginally significant, *F*(1, 59) = 2.60, *p* = 0.08, MSE = 0.004. In sum, although suggested by the data pattern, no strong evidence exists that visually aided CS-US-rehearsal has a more delayed effect on responding than purely mental rehearsal.

### Discussion

The data pattern in the “Visual Rehearsal”-group replicated the findings of Experiments 1 and 2. After rehearsal of the CS+_R_, suppression to the CS+_NR_ decreased significantly stronger than suppression to the CS+_R_. Moreover, responding to the CS+_R_ seemed to persist longer as evidenced by a non-significant decline from post-acquisition to the first rehearsal test, while this decrease was significant for the CS+_NR_. Importantly, the same pattern emerged for the “Mental Rehearsal”-condition. Again, the decrease in responding was significantly stronger for the CS+_NR_ than for the CS+_R_ and responding to CS+_R_ sustained on the first rehearsal test trial. This suggests that the rehearsal effects of Experiments 1 and 2 should not be attributed to the fact that the rehearsal trials simply constitute additional acquisition trials. Rehearsal impacts responding both when the to-be-rehearsed information is externally or internally generated.

Importantly, the crucial Group × Phase × CS-type interaction was significant, demonstrating an effect of rehearsal in both CS-US-rehearsal groups, but not in the “Control”-group. However, three important notes should be made. First, in the “Mental Rehearsal”-condition, responding to both CS+s already differed at the end of the acquisition phase, which is unexpected given that both CS+s underwent the exact same procedure until then. Although responding to the CS+_R_ shows a slower decrease than responding to the CS+_NR_, the CS+_R_ does not evoke significantly more suppression after rehearsal than the CS+_NR_, which might be attributed to this unexpected post-acquisition difference. Second, because of the difference between both rehearsal conditions in baseline responding to the CS+’s the non-significance of the Group (Visual Rehearsal/Mental Rehearsal) × CS-type interaction should be interpreted with caution. Third, in contrast to Experiments 1 and 2, the rehearsal effect is most strongly present for the overall rehearsal test and to a lesser extent for the first test trial only.

The significant decline for both CS+s in the “Control”-condition seems to demonstrate that the natural course of conditioned suppression is to decrease over time, rather than to persist at the same level. This suggests that the decrease in responding to the CS+_NR_ during CS-US-rehearsal in the current and the previous experiments, should not be attributed to some kind of interference from the CS+_R_.

A final important remark is that more participants had to be excluded because of a negative attention score than in the previous experiments. Although the number of participants with a negative score was not significantly associated with the experimental group, χ^2^(2) = 1.033, *p* = *0.60*, this exclusion was especially remarkable in the “Mental Rehearsal”-condition, where 26.92% of the participants were omitted. Presumably, this should be attributed to the less concrete nature of the rehearsal task in this group, where only an exclamation mark was presented.

## General Discussion

In human conditioning studies, relatively little attention has been devoted to the processing of a memory trace after its initial acquisition. In an attempt to explore the role of active post-acquisition processing in conditioning, we experimentally induced repeated activation of a CS-US-contingency memory and tested whether this impacted conditioned responding at test. Experiment 1 showed that rehearsing a previously acquired CS-US-contingency leads to stronger CRs to the rehearsed than to a conditioned, but non-rehearsed CS+. In Experiment 2 this effect was replicated and, in addition, it was shown that no such difference occurred when rehearsal was focused on the CS alone (rather than the CS-US-contingency). Experiment 3 demonstrated that the same rehearsal effect is found regardless of how mental rehearsal is induced. Priming rehearsal through visual presentation of the CS-picture and a verbal reference to the US (as in Experiments 1 and 2) has the same effect as a purely mental procedure. Moreover, results of the “Control”-condition suggest that the natural course of responding to CS+s after acquisition (and during an irrelevant rehearsal task) is a decrement in suppression. An important limitation of Experiment 3 is formed by the post-acquisition differences in responding between the conditions. While the rehearsed and the non-rehearsed CS+ evoked the same amount of responding in the “Visual Rehearsal” and the “Control”-condition, this was not the case in the “Mental Rehearsal”-condition. The reason for this unexpected difference in unclear, but it might complicate interpretation of our findings. However, overall, the data show that repeatedly activating the memory trace of a CS-US co-occurrence impacts subsequent conditioned responding, even in the absence of direct experience with the phenomenological aspects of CS-US-pairings. Interestingly, a recurrent finding is that rather than increasing the level of responding, CS-US-rehearsal results in persistent CRs, while the absence of mental activation causes responding to decline. We will discuss this issue in more detail below.

Based on Experiments 1 and 2 an important question was to what extent the rehearsal manipulation entailed learning processes rather than memory processes. Indeed, the procedure of repeatedly activating the CS-US-contingency might have constituted additional *training* trials, given that the CS-US-contingency was partially presented. However, the results of Experiment 3 showed that purely mental rehearsal, which was not cued by the CS and a verbal reference to the US and was thus internally generated, affected conditioned responding in a similar way.

This issue demonstrates that research on rehearsal effects in conditioning is located at the interface of learning and memory. Two alternative positions exist regarding the interrelation of these two processes. One perspective is that learning occurs only when external input is present, while memory processes pertain to internally generated input. In that case, learning occurred in Experiments 1 and 2, while the rehearsal manipulation in Experiment 3 elicited a memory process, rather than a learning process. An alternative viewpoint is to define learning as a change in behavior due to experience (e.g., Bower and Hilgard, 1981). In this perspective, learning occurred in all three experiments, as the results showed a change in conditioned behavior compared to when no repeated activation was induced. In sum, both learning and memory seem crucial in understanding conditioning effects (cf. Bouton and Moody, 2004) and their interplay is an important but generally ignored topic.

The current research ties up with an increasing body of research demonstrating that mental representations of a conditioning stimulus can influence conditioned responding in the absence of the physical stimulus. An overview of this literature is provided by Dadds et al. (1997), Holland (1990), and Pickens and Holland (2004). In short, there are two important lines of evidence for the fact that activation of the mental representation of a stimulus can induce learning to that stimulus. First, in US-revaluation studies it is found that conditioned responding decreases/increases after devaluation/inflation of the US without directly experiencing the CS-US-contingency (e.g., Rescorla, 1974; White and Davey, 1989; de Jong et al., 1996). Second, in studies on representation-mediated learning (Holland, 1990; Pickens and Holland, 2004) it is typically shown that an associatively activated stimulus representation can substitute for actually presented stimuli. Besides demonstrating that an association may be formed with a stimulus when the stimulus is not presented, it is also demonstrated that associations may be formed between two stimuli even when *both* of the stimuli are absent, rather than only one of them (e.g., in animals: Holland and Sherwood, 2008; in humans: Le Pelley and McLaren, 2001).

Besides the theoretical importance of our results in bringing research traditions on memory and learning closer together, the idea of mental rehearsal is clinically relevant as well. Overall, the data indicate that not only the acquisition experiences themselves, but also the way in which one cognitively engages in the memories of these events, has an impact on conditioned responding. Clinical observations suggest that individuals differ in their tendency to engage in repetitive thought such as worry and rumination. Repetitive thought is defined as “the process of thinking attentively, repetitively, or frequently about one’s self and one’s world” (Segerstrom et al., 2003, p. 909). Hence, this variable can be considered as a form of active rehearsal. After experiencing a traumatic event, rehearsal of this negative event together with associated stimuli might strengthen the acquired CS-US-association. Previous work by Otto et al. (2007), as well as a more recent study in our laboratory (Joos et al., 2012c), points in that direction. Otto et al. (2007) found trait worry to be a good predictor of the strength of fear acquisition. We replicated the finding that individuals with a higher level of worry demonstrated more pronounced fear acquisition. Moreover, this association could not be explained by trait anxiety (Joos et al., 2012c). One way to explain this relation between worry and conditioning strength is that the high trait-worriers mentally repeat the CS-US-contingency during acquisition and therefore show stronger conditioned responding in the fear conditioning task. Of course, post-acquisition mental repetition of the fear memory is only one route that might play a role. As such, these studies do not provide direct evidence of the impact of rehearsal after acquisition and differ in this respect from the experimental studies presented in this paper.

Given the conclusion that post-acquisition rehearsal impacts conditioned responding, an important question pertains to the exact processes that are responsible for this effect. A first candidate in explaining the results is *consolidation*, which refers to the progressive post-acquisition strengthening of memory traces in long-term memory (Dudai, 2004). Repeated activation of the CS-US-contingency might strengthen the association between the mental representations of the CS and the US in memory, resulting in a cognitive consolidation of this memory trace.

A second mechanism focuses on the decrease in conditioned responding to the non-rehearsed CS+. As suggested before, the CS-US-rehearsal trials might *interfere* with responding to the non-rehearsed CS+ at test. However, the results of the “Control”-condition of Experiment 3 indicate that the recurrently observed decrease in responding to the non-rehearsed CS+ is the natural course of responding and should therefore not be attributed to interference by a related stimulus (CS+_R_), as the same reduction in CRs is evident when no related stimulus was rehearsed.

That the natural course of conditioned suppression after rehearsal is to decrease, rather than to persist at the same level supports the likelihood of a third possible underlying mechanism, i.e., *prevention-of-forgetting*. Indeed, this decrease might be considered as a type of forgetting. Hence, mentally rehearsing a CS-US-contingency might prevent forgetting of this memory trace through repeated activation. This idea is in line with the notion that rehearsal prevents the loss of information in short-term memory (e.g., Atkinson and Shiffrin, 1971; Portrat et al., 2008). More specifically, as forgetting in conditioning probably relates to a lack of accessibility of the memory trace rather than a loss of information (Anderson, 2000; Bouton, 2004), rehearsal might counteract a spontaneous decrease in accessibility of the memory trace. In all three reported experiments, we see a decrease in CR strength to the non-rehearsed CS+ between acquisition and test, supporting the claim that participants “forget” this association to some extent. For the “CS-Rehearsal”-group in Experiment 2, conditioned responding to both CS+s also decreases between acquisition and test, but this decrease fails to reach statistical significance on the first test trial. However, the non-significant Group × Phase (Acquisition test/Rehearsal test 1) interaction for the non-rehearsed CS+ suggests that both groups show the same decreasing pattern of responding to the non-rehearsed CS+. Overall, our data seem to support the notion that rehearsal renders the CS-US-memory more accessible, resulting in stronger CRs upon subsequent CS-presentations compared to presentations of the non-rehearsed CS.

In conclusion, the present studies show that repeated post-acquisition activation of a CS-US-contingency sustains conditioned responding. Through experimental induction of post-acquisition CS-US-repetition, it was shown that active post-encoding processes such as rehearsal, which are frequently studied in the memory literature, might play an important role in conditioning as well. In particular, long-term conditioning effects may be largely influenced by such memory processes. Additionally, our results indicate that individual differences in the tendency to reflect upon past experiences, as in worry or rumination, might create differences in conditioned responding, due to varying levels of post-acquisition activation of the CS-US-memory.

## Conflict of Interest Statement

The authors declare that the research was conducted in the absence of any commercial or financial relationships that could be construed as a potential conflict of interest.

## Footnotes

^1^In Experiment 1, the Penn State Worry Questionnaire – Past Day (PSWQ-PD; Joos et al., 2012a) and the Sensitivity for Punishment-Sensitivity for Reward-questionnaire (SPSRQ; Torrubia et al., 2001) were administered at this stage. In Experiment 2, the Penn State Worry Questionnaire (PSWQ; Meyer et al., 1990), the Mindfulness Attention Awareness Scale (MAAS; Brown and Ryan, 2003), and the Questionnaire upon Mental Imagery (QMI; Sheehan, 1967) were administered. In Experiment 3, the MAAS and the PSWQ-PD were filled out. There were no significant associations between the questionnaire scores and the effect of rehearsal on conditioning performance. These null findings might be attributed to the fact that the rehearsal manipulation in the current studies overruled the effect of individual difference variables on the strength of the conditioning response.

^2^In all 3 three experiments, data were first analyzed using an ANOVA with CS-type (CS−-_A_, CS−-_B_, CS+_R_, and CS+_NR_) as within-subjects variable (and Group as between-subjects variable in Experiments 2 and 3). Given that in every group of participants the SR’s did not differ between both CS−‘s, SRs for both stimuli were always averaged for use in subsequent analyses.
